# Supplementary figures and images for: Prevalence of Mycobacterium leprae in armadillos in Brazil: A systematic review and meta-analysis
Source: PLoS Negl Trop Dis. 2020 Mar 23;14(3):e0008127. doi: 10.1371/journal.pntd.0008127 (PMC7156091; doi:10.1371/journal.pntd.0008127)

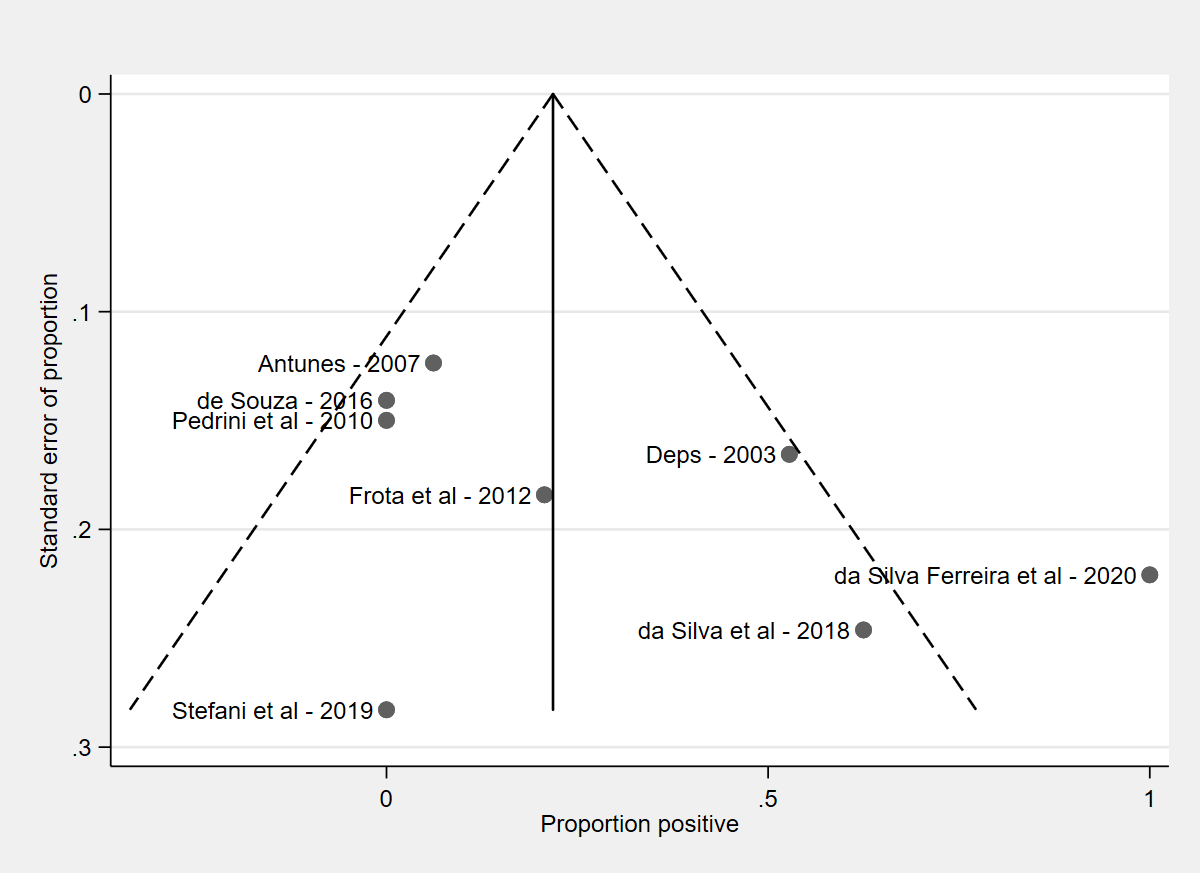

Supplement: S1 Fig — (TIF) [file pntd.0008127.s002.tif]

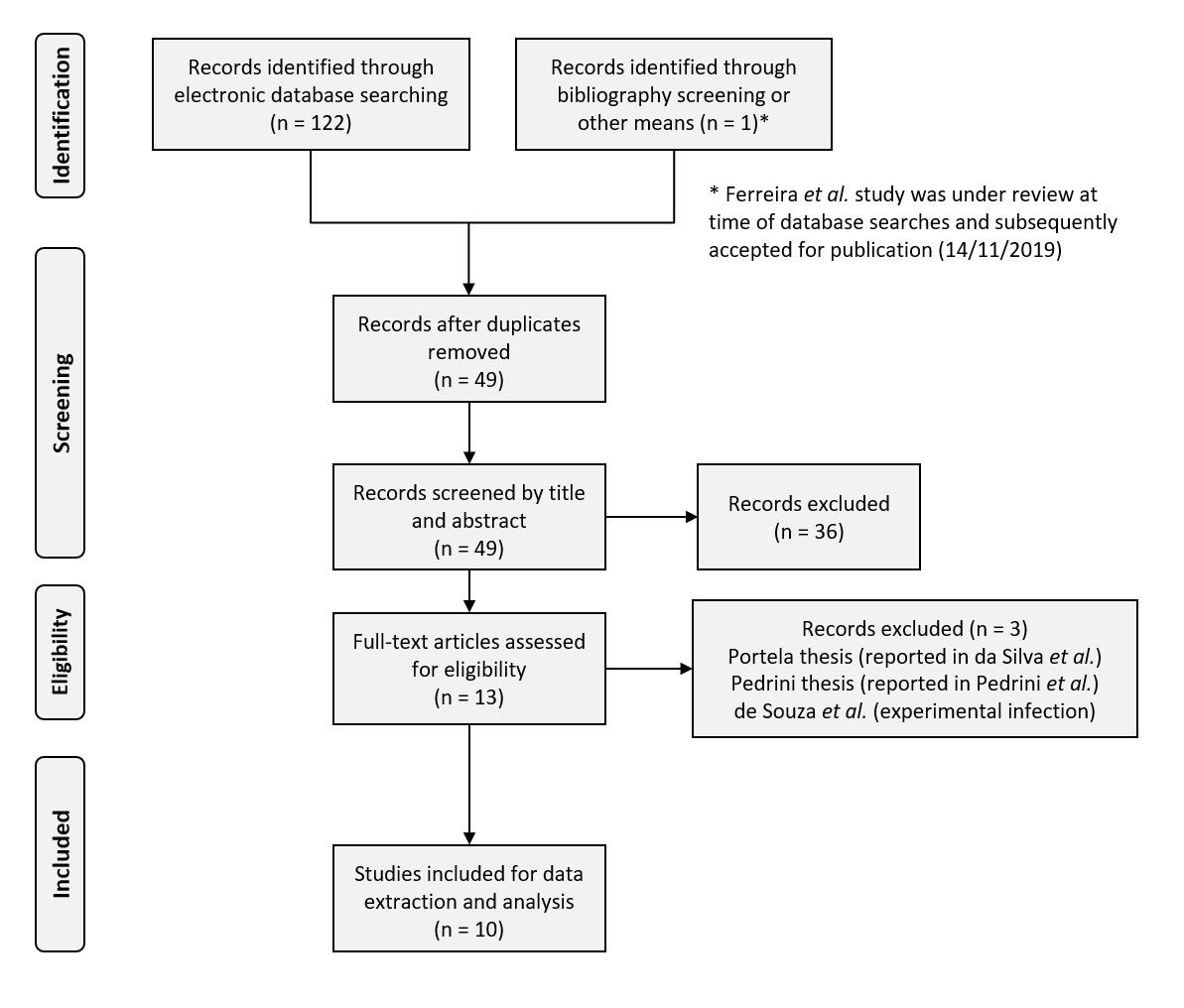

Supplement: S1 Flow Diagram — (TIF) [file pntd.0008127.s005.tif]
