# Supplementary material for: Prevalence of Mycobacterium leprae in armadillos in Brazil: A systematic review and meta-analysis
Source: PLoS Negl Trop Dis. 2020 Mar 23;14(3):e0008127. doi: 10.1371/journal.pntd.0008127 (PMC7156091; doi:10.1371/journal.pntd.0008127)
Supplement: S1 Table — (DOCX) [file pntd.0008127.s003.docx]

**Table S1: Detailed description of included studies investigating *M. leprae* infection in wild armadillos in Brazil**

| **Author and year** | **State(s)** | **Methods** | **Results** |
| --- | --- | --- | --- |
| Deps 2003 | Espírito Santo | PhD thesis, with preliminary PCR findings reported in Deps *et al.* 2002, PGL-1 rapid immunochromatographic serology in Deps *et al.* 2007 and PGL-1 ELISA results in Deps *et al.* 2008. Full PCR results are reported for the first time in this systematic review. | Of the 52 armadillos captured, 27 were male and 25 were female. Weight ranged from 350-5200g (median 1428g). All were *Dasypus novemcinctus*. 23 armadillos died during transport or whilst in captivity, thereby providing organ tissue samples (brain, spleen, kidney, lung, liver, and intestine). |
|  |  | 52 armadillos were captured between June 2000 and July 2001 from the rural area of six municipalities: Serra (Private Environmental Reserve-CST), n=43; Cariacica (Pedro Fontes Leprosy Colony), n=3; Guarapari, n=3; Muniz Freire, n=1; Vila-Velha n=1; and C. do Itapemirim, n=1 | 6 armadillos had body or head ulcers – none had a typical clinical picture of armadillo Virchowian leprosy (‘leprosy’ skin lesions). 50 had ulcerated lesions on the paws and/or internal carapace; one armadillo had detectable lymphadenomegaly on palpation in the inguinal region. |
|  |  | Skin and ear tissue samples were collected under anaesthesia from apparently healthy animals. Half of the samples were stored at -20°C for use in PCR, the other half in 10% formalin solution for histopathological examination. | **Combined *M. leprae* prevalence by PCR in tissues and/or anti-PGL-1 in blood was 46% (24/52); 4 armadillos were positive for both tests. Concordance between tests was 89% for skin PCR and lesion PCR and 88% for ear PCR and ELISA.** |
| Deps *et al.* 2007 | Espírito Santo | Armadillo sampling described in Deps 2003. The ML Flow test was performed on whole blood or serum samples from 37 animals in accordance with the method described by Buhrer-Sekula *et al.* 2003 | **The ML Flow test gave positive results in 11/47 armadillos (30%) (+2 in 4 animals, +1 in 7 animals)** |
| Deps *et al.* 2008 | Espírito Santo | Armadillos sample as described in Deps 2003 but with an additional 14 armadillos (total 66) of which serum samples for ELISA were available from 47 armadillos. | **Anti-PGL-1 antibodies were detected in 5/47 armadillos (10.6%)** |
|  |  | ELISA was performed in accordance with the method described by Brett *et al.* 1986. | Among the 5 armadillos positive by ELISA, 4 presented ulcers and/or nodules of which 1 armadillo also had enlarged inguinal lymph nodes. |
| Antunes 2007 | Espírito Santo | MSc dissertation, with related findings published in Antunes *et al.* 2009. PCR results are reported for the first time in this systematic review. | Of the 69 armadillos captured, 31 were male and 38 were female. Weight ranged from 400-6000g. All were *Dasypus novemcinctus*. |
|  |  | Armadillos were captured professionally in the rural areas of 4 municipalities between July 2004 and July 2005: Marechal Floriano, n=3; Serra, n=5; Vitória, n=3; and Alegre, n=48. | ***M. leprae* DNA was found in 6% (4/65) of armadillos tested by PCR with RLEP primer. All RLEP PCR-positive animals showed at least one clinical alteration.** |
|  |  | After blood collection and clinical examination, armadillos were sacrificed under anaesthesia by exsanguination. Tissue samples were collected during autopsy (brain, lung, heart, tongue, skin, ear, kidney, spleen, liver, intestine, testis, uterus and cervical, inguinal and mesenteric lymph nodes) and stored in 10% buffered formaldehyde, 70% alcohol at -4°C for use in PCR. | Clinical alterations such as external lesions, lymphadenomegaly, liver and splenic lesions, and visceromegaly were identified in 95% of the armadillos. All 18kDa primer PCR results were negative, but 2 animals were positive by RT-PCR for RLEP and 18Kda primers. DNA from samples of these 2 animals was sequenced and confirmed as repetitive sequence RLEP copies 4 and 6, which are specific to *M. leprae*. |

| **Author and year** | **State(s)** | **Methods** | **Results** |
| --- | --- | --- | --- |
| Pedrini *et al.* 2010 | São Paulo and Mato Grosso do Sul | 44 wild armadillos of four species were captured from the Middle Western area of the state of São Paulo, in the municipalities of Botucatu, Pardinho, São Manuel, Manduri, and Bauru, and from the Nhumirim ranch, an experimental station of Embrapa Pantanal, located in the Pantanal da Nhecolândia of Mato Grosso do Sul state (undated). | Of the 44 armadillos captured, 27 were male and 17 were female. Weight ranged from 3500 to 6500g. Species were: *D. novemcinctus,* n=18; *E. sexcintus,* n=22; *C. tatouay,* n=2; *C. unicinctus,* n=2. |
|  |  | 138 samples were obtained, comprising: 26 ear fragments, 32 faeces, 21 nostril swabs, 20 blood samples, and 39 internal organ samples (liver 15, spleen 7, lymph node 10, kidney 1, adrenal gland 1, lung 2) | **No mycobacteria were visualized in the organ samples submitted to histopathological examination and bacilloscopy and all PCR reactions were negative for *M. leprae.*** |
| Frota *et al.* 2012 | Ceará | 29 wild armadillos from two species were captured by local hunters between July-August 2007 by local hunters under the supervision of a veterinarian in rural sites of 12 selected endemic municipalities from Ceará. | Weight ranged from 2600-3800g. Species were: *D. novemcinctus*, n=27; *E. sexcinctus*, n=2. |
|  |  | 116 liver, spleen, ear and nose tissue samples were tested using a nested RLEP PCR assay. The primers for RLEP2-1 and RLEP2-2 amplified a 282-bp sequence of the RLEP element. The second set of inner primers, RLEP2-3 and RLEP2-2, amplified a 238-bp product. The *M. leprae* gyrA region was amplified using primers gyrAF and gyrAR. | ***M. leprae* was detected in 6/29 (21%) animals; 5 *D. novemcinctus* and one *E. sexcinctus*. *M. leprae* DNA was amplified in the ear biopsy samples of all 6 animals, but in only 5 of the liver or nose biopsy samples and 3 of the spleen biopsy samples.** |
| Kluyber 2016 | Mato Grosso do Sul | MSc dissertation. 50 wild armadillos from four species were sampled, 43 captured live from Pantanal and 7 found dead on roads in the Cerrado ecoregion of Mato Gross do Sul between June 2011 and January 2015. | Species were: *P. maximus*, 16; *E. sexcinctus*, 23; *D. novemcinctus*, 3; C. unicinctus, 8. |
|  |  | Blood and ear tissue samples were taken from anaesthetised live animals, and lung, liver and spleen samples from roadkill animals. PCR with RLEP2-1/RLEP2-2 primers, plus TaqMan® qPCR and multiplex PCR of multiple-locus variable number tandem repeat (VNTR) sequences. Sequencing to determine *M. leprae* SNP type (1-4) and subtype (1A-4P) and WGS mentioned in methods. | **Tentatively positive real time and multiplex PCR results for samples from 4 animals not confirmed in conventional PCR, which returned negative results for all samples tested.** |
| da Silva *et al.* 2018 | Pará | 16 armadillos were captured by locals hunting in the tropical forest surrounding two communities on the Santarém-Cuiabá highway from the municipality of Belterra in western Pará state: São Jorge (92km) and Corpus Christi (135km). | Average weight of armadillos was 3700g. All were *Dasypus novemcinctus*. |
|  |  | Samples of armadillo liver and spleen tissue (minimum 1cm^3^) were obtained from freshly killed animals from different in both villages: 3 from Corpus Christi, 13 from São Jorge. Samples were placed in 5ml plastic tubes containing 70% ethanol for Qiagen® multiplex PCR using RLEP LP1 forward and LP2 reverse primers and staining to detect mycobacterial DNA, cell wall constituents and *M. leprae* PGL-1. | **Extracted DNA from spleen tissues of 10/16 (62%) armadillos tested positive for the presence of the RLEP repetitive sequence by PCR. Of 5 armadillos examined from the same group, armadillos that were RLEP positive in the spleen were also RLEP positive in the liver while animals that were RLEP negative were negative in both. Signal strength in positive animals was much stronger in spleen than in liver.** |
| **Author and year** | **State(s)** | **Methods** | **Results** |
| Stefani *et al.* 2019 | Amazonas | 12 wild young adult armadillos caught in a rural hyper endemic leprosy area of Coari municipality located by the Solimões River between the Mamiá and Coari Lakes, 444km from Manaus. Local inhabitants caught armadillos in cages which were brought to the expedition boat where they were anesthetized before being euthanized by exsanguination. A pilot expedition took place in June 2015, the main expedition in August 2015. | Weight range was 4000-5000g. All were *Dasypus novemcinctus*. None of the armadillo’s tissue sections examined was positive for bacilli detection by FF, whilst HE stained tissue sections did not show histopathological features of *M. leprae* infection except for one skin fragment that presented unspecific inflammatory infiltrate suggestive of indeterminate leprosy. PAS staining for fungal infection was negative in skin sections presenting granulomas, foreign-body granuloma, and non-granulomatous chronic dermatitis. |
|  |  | Armadillo tissue fragments (skin, spleen, livers, lymph nodes, adrenal glands, ovary, fallopian tube) were prepared for histopathological examination after staining with HE and FF and for *M. leprae* RLEP qPCR (Qiagen®) (148bp). Animal tissues presenting granulomas or non-granulomatous dermatitis were examined for the identification of fungus after periodic acid–Schiff (PAS) staining. 96 slides were prepared from 48 specimens for histopathology, with a median 8 slides examined per animal (ranging from 4 to 12 slides). | ***M. leprae*-specific qPCR for RLEP gene sequence was negative in all armadillo tissue samples tested. *M. leprae* Thai 53 DNA added to negative PCR samples resulted in amplification of the RLEP amplicon in all extracts demonstrating that DNA samples from armadillos did not contain inhibitory substances.** |
| Ferreira *et al.* 2019 | Rio Grande do Norte | 20 wild armadillos caught by local hunters from 20 distinct locations within five rural municipalities (Pendências, Afonso Bezerra, Macau, Pedro Avelino e Guamaré), 10 animals in May 2016, 10 in June 2016. | Of the 20 armadillos captured, 11 were male and 9 were female. Mean weight was 1645g. All were *E. sexcinctus.* Clinical examination detected the presence of skin lesions in 6 animals (30%), splenomegaly in 4 (20%) and lymphadenopathy in 7 (35%). |
|  |  | Blood samples were taken from anaesthetised animals which were then euthanized by administration of potassium chloride *via* the femoral vein. Samples of liver were collected for molecular analyses during necropsy. Serum was examined using two in-house ELISAs and two commercially available immunochromatographic lateral flow tests (ML flow and NDO-LID®) for detection of PGL-1 and/or LID-1 *M. leprae* antigens. *M. leprae*-specific repetitive element (RLEP) was the target in conventional and nested PCR assays in liver tissues. | **Anti-LID-1 ELISA positivity was 5% (1/20); 17/20 (85%) of sera tested by NDO-LID and 16/20 (80%) by ML Flow rapid test were scored as positive. PCR and anti-PGL-1 ELISA positivity was 100%.** |

**References**

Antunes JM. 2007. Pesquisa de Mycobacterium leprae em tatus selvagens da espécie Dasypus novemcinctus do Estado do Espírito Santo [Mestrado]. VITÓRIA: UNIVERSIDADE FEDERAL DO ESPÍRITO SANTO.

Antunes JM, Zanini MS, Demoner LdC, Deps PD. 2009. Diagnosis of Mycobacterium leprae in armadillos (Dasypus novemcinctus) and the correlation with water source proximity in Rive county, Espírito Santo State-Brazil. / Diagnóstico de Mycobacterium leprae em tatus (Dasypus novemcinctus) e sua correlação com a proximidade das fontes de água no distrito de Rive, Espírito Santo-Brasil. Veterinária e Zootecnia. 16(4):642-649.

Brett SJ, Payne SN, Gigg J, Burgess P, Gigg R. 1986. Use of synthetic glycoconjugates containing the Mycobacterium leprae specific and immunodominant epitope of phenolic glycolipid I in the serology of leprosy. Clinical and experimental immunology. 64(3):476-483. eng.

Buhrer-Sekula S, Smits HL, Gussenhoven GC, van Leeuwen J, Amador S, Fujiwara T, Klatser PR, Oskam L. 2003. Simple and fast lateral flow test for classification of leprosy patients and identification of contacts with high risk of developing leprosy. J Clin Microbiol. 41(5):1991-1995. eng.

da Silva MB, Portela JM, Li W, Jackson M, Gonzalez-Juarrero M, Hidalgo AS, Belisle JT, Bouth RC, Gobbo AR, Barreto JG et al. 2018. Evidence of zoonotic leprosy in Para, Brazilian Amazon, and risks associated with human contact or consumption of armadillos [Article]. PLoS Negl Trop Dis. 12(6):e0006532.

Deps PD. 2003. Pesquisa de *Mycobacterium leprae* em tatus selvagens da espécie *Dasypus Novencintus* do Estado do Espírito Santo [Doutorado]. São Paulo: Universidade Federal de São Paulo.

Deps PD, Antunes JM, Faria C, Buhrer-Sekula S, Camargo ZP, Opromola DV, Tomimori J. 2008. Research regarding anti-PGL-I antibodies by ELISA in wild armadillos from Brazil [Article]. Rev Soc Bras Med Trop. 41 Suppl 2(SUPPL. 2):73-76.

Deps PD, Antunes JM, Tomimori-Yamashita J. 2007. Detection of Mycobacterium leprae infection in wild nine-banded armadillos (Dasypus novemcinctus) using the rapid ML Flow test [Article]. Rev Soc Bras Med Trop. 40(1):86-87.

Deps PD, Santos AR, Yamashita-Tomimori J. 2002. Detection of Mycobacterium leprae DNA by PCR in blood sample from nine-banded armadillo: preliminary results. Int J Lepr Other Mycobact Dis. 70(1):34-35. eng.

Frota CC, Lima LN, Rocha Ada S, Suffys PN, Rolim BN, Rodrigues LC, Barreto ML, Kendall C, Kerr LR. 2012. Mycobacterium leprae in six-banded (Euphractus sexcinctus) and nine-banded armadillos (Dasypus novemcinctus) in Northeast Brazil [Article]. Mem Inst Oswaldo Cruz. 107 Suppl 1(SUPPL.1):209-213.

Kluyber DdS. 2016. Avaliação da Prevalência de Patógenos Zoonóticos de Importância para Saúde Pública em Tatus de Vida Livre – Mato Grosso do Sul – Brasil [Mestrado]. UNIVERSIDADE DE SÃO PAULO.

Pedrini SC, Rosa PS, Medri IM, Mourao G, Bagagli E, Lopes CA. 2010. Search for Mycobacterium leprae in wild mammals. Braz J Infect Dis. 14(1):47-53.

Stefani MMA, Rosa PS, Costa MB, Schetinni APM, Manhães I, Pontes MAA, Costa P, Fachin LRV, Batista IMFD, Virmond M et al. 2019. Leprosy survey among rural communities and wild armadillos from Amazonas state, Northern Brazil. PLoS ONE. 14(1):e0209491-e0209491.
