# Supplementary material for: Prevalence of Mycobacterium leprae in armadillos in Brazil: A systematic review and meta-analysis
Source: PLoS Negl Trop Dis. 2020 Mar 23;14(3):e0008127. doi: 10.1371/journal.pntd.0008127 (PMC7156091; doi:10.1371/journal.pntd.0008127)
Supplement: Attachment — Submitted filename: Review Deps meta-analysis_2.docx [file pntd.0008127.s006.docx]

Prevalence of *Mycobacterium leprae* in armadillos in Brazil: a systematic review and meta-analysis

Reviewer: Ligia Kerr

This paper is well written and related to an important health problem in Brazil: leprosy. The objectives of the authors were to characterize studies which have investigated natural *M. leprae* infection in wild armadillos in Brazil, and to quantify and explore variability in the reported prevalence of infection.

This systematic review of Brazilian scientific papers, together with other studies carried out in other regions of the world, points out that armadillos may be potential transmitters of *M. leprae* to humans since they can be infected by *M. leprae*. The papers reviewed are appropriate and the authors also included unpublished theses and dissertations.

The authors raise the issue that PCR tests that do not take potential inhibitors into account may result in false negative tests, a fact that could justify the low levels of positivity in some of the studies reviewed. A second problem which the authors recognize is the small sample size used in several papers which could underrepresent seropositivity. I need to comment that the papers reviewed cover widely different environments with huge differences in flora and fauna. The large confidence intervals around their estimates are discussed well in the paper. Variability of the prevalence among armadillos can be related not only to different environments but also great diversity in the prevalence of leprosy in these areas in Brazil. Brazil is a continent size country with high urbanization and uneven population density in rural areas. In urban areas contacts between humans and armadillos would be rare. A hypothesis for zero prevalence among armadillos in Central-West Region could be the Pantanal that floods during raining season. This environment favors high populations of wild animals and larger numbers of armadillos and representative samples would require many more animals to represent the true prevalence of leprosy among armadillos.

The authors might also consider discussing that the transmission between armadillos and humans could be other way around. Wild armadillos from Louisiana, which are naturally infected with *M. leprae* were found to harbor the European/North African SNP type 3 strain, indicating that they were infected from human sources (Monot M, Honoré N, Garnier T, Araoz R, Coppee JY, et al. (2005) On the origin of leprosy. Science 308: 1040–1042).

An important recommendation raised by the authors is to standardize methods with a protocol based on best practice in specimen collection and simpler and more rapid methods such blood testing for *M. leprae*, rather than taking tissue samples from sacrificed animals. However such tests have lower sensitivity. I am sympathetic with this proposal since armadillos are animals declining in the wild and efforts should be made to preserve them. However, comparability with other studies would be much more complex or even impossible.

A paper reporting a study of human-armadillo interaction among residents of the State of Ceará, in northeastern Brazil, shows a great variety of contact with armadillos either through hunting, through cooking, or through consumption of armadillo meat. This study raises important and relatively unreported issues about contact between human beings and armadillos that the authors could cite. The authors demonstrate evidence of numerous situations in which leprosy transmission via armadillo is possible (Kerr et al. *Human–armadillo interaction in Ceará, Brazil: Potential for transmission of Mycobacterium leprae*. **Acta Tropica**. 152: 74-79, Dec 2015).

Finally, the authors do appropriately note how difficult it is to evaluate the role of the armadillo in human transmission, still, acknowledging the possible role for the armadillo is an advance in a field that has steadfastly resisted such conjectures.

Conclusion: I found the paper well-written and informative, with the caveats surrounding drawing a single general seroprevalence level for Brazil from studies in such varied environments.
