# Supplementary material for: Prevalence of Mycobacterium leprae in armadillos in Brazil: A systematic review and meta-analysis
Source: PLoS Negl Trop Dis. 2020 Mar 23;14(3):e0008127. doi: 10.1371/journal.pntd.0008127 (PMC7156091; doi:10.1371/journal.pntd.0008127)
Supplement: Attachment — Submitted filename: Responses to Reviewers Comments.docx [file pntd.0008127.s007.docx]

**Responses to Reviewers’ Comments**

1. Reviewer #3: Investigate natural M leprae infection in wild armadillos in endemic country to disease in human that have a control program but the new cases are maintenance and as armadillos are eaten in theses areas they can be infection to human and human to armadillos but quantify and explore the variability in prevalence of armadillos infection will be very difficult with this systemic review.

**The variability is determined by the data – the challenge is to interpret the observed variability. We think that our manuscript, with amendments in response to specific peer reviewer comments, has risen to this challenge by discussing the most plausible explanations for the observed variability.**

2. Reviewer #3: It is a systematic review protocol, but there are many limitations by the variability of methodology between the studies.

**As in our response to this reviewer’s first comment, we have discussed variation in methodology as a limitation and possible source of variability, although as we also mention, the meta-analysis indicates that this is also consistent with sampling error (1^st^ paragraph of Discussion).**

3. Reviewer #3: The armadillos live in endemic areas of hansen disease human and are animals susceptible to infection of M leprae, but as they no are domestic animals perhaps all sample had few size.

**Relatively small sample sizes were indeed a limitation, as mentioned in our Discussion in relation to explaining the observed variation (1^st^ paragraph of Discussion).**

4. Reviewer #3: The authors suggest new study only with blood and in few studies the armadillos were euthanised and probable when ethical or regulatory requirements were more light to experimental in animals.

**Indeed, and our Discussion suggests that non-lethal approaches are preferable, regardless of ethical and regulatory requirements.**

5. Reviewer #2: The analysis matches the plan, the results are clearly presented, tables have clarity but images are of low quality, suggest replacement.

**PLoS uses low-quality images in PDF for peer review purposes. Uploaded images are higher resolution.**

6. Reviewer #3: line 113-4: M leprae was detected is important defined what is this? Same line 127: prevalence of M leprae.

**We have reworded the definition on lines 114 as “captured armadillos which tested positive for M. leprae” and inserted “(% positive armadillos)” on line 128.**

7. Reviewer #3: Table 1 is better figure 1:

**Figure 1 is intended to be information for readers who are unfamiliar with the geographical locations of the states in Brazil and to illustrate the geographical coverage of the included studies.**

8. Reviewer #3: Deps 69 or 66 cases included 52 need more explain about this. line 151-3

**In the first part of this sentence (lines 151-152) we have inserted the author names (Deps and Deps *et al.*) and the word ‘total’ to clarify that the 66 and 52 armadillos were part of a larger sample (of 66 animals) caught by these authors, and in the second part of the sentence we have inserted the name of the author (Antunes) corresponding to the sample of 69 armadillos and the word ‘later’ to make it clear that this was a different sample.**

9. Reviewer #3: Antunes line 202 (2 or 4 /65)

**The given proportion (2/65) is correct in this sentence, corresponding to 18kDa (non-RLEP) PCR. The same study reported 4/65 for RLEP PCR (line 199).**

10. Reviewer #3: Frota 27 + 2 = 2

**We are unsure what this comment refers to – we report Frota’s results as the proportion positive 6/29 in a total sample comprising 27 *D. novemcinctus* + 2 *E. sexcinctus*.**

11. Reviewer #1: To some extent, yes. The conclusion clearly state the main finding in what relates the proposed objective. However, some digression to the main objective seems to be unnecessary and somewhat tendentious to a subject leprosy as a zoonotic disease) not strictly relate to the main objective (see below).

**We have made revisions in response to specific comments on this issue, as detailed point-by-point below.**

12. Reviewer #3: Objectives of quantify and explore the variability in prevalence of armadillos infection will be very difficult with this systemic review, because of the limitations by the differences in methodology of the studies and because the definition of infection in leprosy is yet very difficult if the same methodology is used, excepted when the bacillos are found in tissue for stain specific but few studies used this methodology.

**As in our response to this reviewer’s earlier comment, which is entirely valid... the variability is determined by the data, the challenge is to interpret the observed variability. We think that our manuscript has met this challenge by discussing the most plausible explanations for the observed variability, including sampling error. Further, the main meta-analysis used data from studies which used the same methodology (RLEP PCR). This does not entirely remove differences in methodology as a source of variability, as we discuss at length in our Discussion.**

13. Reviewer #1: L. 72 - …sequelae of Hansen’s Disease are entirely avoidable if diagnosed and treated early [13]… I would suggest to remove “entirely” – sometimes this is not true.

**We have removed the word ‘entirely’.**

14. Reviewer #1: L. 74-77 - Whilst the proportion of new cases in Brazil attributable to zoonotic transmission is unknown, the persistence of armadillo capture and consumption as a cultural habit in some parts of Brazil means that the role of wild armadillos as a reservoir of M. leprae is relevant to efforts to eliminate the disease [16]. This paragraph has an intrinsic disruption. There is no sense to link “the persistence of armadillo capture and consumption” to the possibility of armadillos being or not a reservoir of M. lerprae and, chiefly, to link this to efforts to eliminate leprosy! This paragraph is equivocal and should be deleted.

**We have retained this sentence but reworded it to remove the link to ‘efforts to eliminate the disease’. Instead we simply state that “armadillo capture and consumption persists as a cultural habit in some parts of Brazil and wild armadillos in these areas may serve as a reservoir of *M. leprae* infection in humans.”**

15. Reviewer #3: the bacillos are found in tissue for stain specific but few studies used this methodology explain more about this limitations, or the definition of infection.

**The histopathological results were secondary to our main analysis and insufficient to support a meta-analysis. We therefore decided to summarize them qualitatively, and we describe them in our Discussion as being “less useful” and “difficult to perform in the field”. We have added that the discordance between HE & ZN results and PCR results demonstrates the higher sensitivity of the latter method.**

16. Reviewer #1: Indeed, difficult to ascertain how much the variability in prevalence might be attributed to differences in methods or how much it represents a real variation in the prevalence of M. leprae in the armadillos. This reported limitation recommends some extra caution in their conclusions: leprosy as a zoonosis in USA is a somewhat "political" declaration and to me it is a wise decision of the Brazilian health authorities not to take this seriously for Brazil. It seems that authors do not agree with this cautious and correct decision - however, the results of their review paper is in itself a strong reason for not doing so!

**We have altered some of the wording in our conclusion, changing the prevalence of *M. leprae* in armadillos from being a “key factor” to “possible factor”. With regard to health authorities in Brazil, we have changed the wording to observe that “no recommendations have been made” without casting judgement. However, we have removed the word “appropriate” from the preceding sentence about recommendations in the USA, stating simply “recommendations regarding contact” and we have changed “have been forthcoming” to “have been made”.**

17. Reviewer #1: Authors should be congratulated for intensively recommend new and lengthy studies in the attempt to correlate M. leprae in wild armadillos with proximity to human habitats. Meanwhile, considering their results & conclusions, let us not blame armadillos for playing any key role in the epidemiology of leprosy in Brazil – there are no clear evidences for that so far. In this sense, we would recommend authors to review some parts of their text (namely in Introduction and Conclusions) where a tendency to speak in favor of this thesis is strongly biased and intentionally suggested. Their main objective is to discuss prevalence of M. leprae in wild armadillos, not to claim that armadillos are responsible for the endemy of leprosy in Brazil, as they tend to suggest.

**We agree that the research question was about the prevalence of *M. leprae* in armadillos, but the motivation of the review was the risk to human health of a zoonotic reservoir of infection. But we have reworded the concluding sentence of the Abstract to remove slightly the emphasis.**

18. Reviewer #2: The authors could discuss in some more considerable depth both the implications of their small sample size, and the great variability of prevalence among the armadillos from the different studies.

**In our statement of findings in the first paragraph of the Discussion we state that “relatively small sample sizes in all the included studies meant that the observed overall variation in *M. leprae* prevalence could be entirely attributable to sampling error, rather than to real variation i.e. some armadillo populations being heavily infected with *M. leprae* whilst others are disease-free or to artefactual variation, i.e. arising from differences in methods.” However, we have extended our discussion of variability in response to comments by Reviewer LK (point #20).**

19. Reviewer #2: The authors fail to cite a study that mentions the possibilities of transmission between armadillos and humans. They also fail to mention/cite that the transmission may occur in the opposite direction as well.

**We cited 5 studies describing risk to humans of transmission from armadillos (references 1-5), and we have added a sixth in response to comment #23 by Reviewer LK (see below). We have also added a sentence mentioning that “The complexities of human-armadillo interaction in relation to M. leprae include the suggestion that transmission can occur in the opposite direction”, citing Monot *et al.* as suggested by Reviewer LK (comment #21).**

20. Reviewer LK: The authors raise the issue that PCR tests that do not take potential inhibitors into account may result in false negative tests, a fact that could justify the low levels of positivity in some of the studies reviewed. A second problem which the authors recognize is the small sample size used in several papers which could underrepresent seropositivity. I need to comment that the papers reviewed cover widely different environments with huge differences in flora and fauna. The large confidence intervals around their estimates are discussed well in the paper. Variability of the prevalence among armadillos can be related not only to different environments but also great diversity in the prevalence of leprosy in these areas in Brazil. Brazil is a continent size country with high urbanization and uneven population density in rural areas. In urban areas contacts between humans and armadillos would be rare. A hypothesis for zero prevalence among armadillos in Central-West Region could be the Pantanal that floods during raining season. This environment favors high populations of wild animals and larger numbers of armadillos and representative samples would require many more animals to represent the true prevalence of leprosy among armadillos.

**We thank the reviewer for the interesting detail in this comment, which we have used to extend our discussion of variation in the prevalence of *M. leprae* infection in armadillo populations in Brazil as a real natural phenomenon by adding the hypothesis about flooding in the Pantanal and the larger animal populations in this area.**

21. Reviewer LK: The authors might also consider discussing that the transmission between armadillos and humans could be other way around. Wild armadillos from Louisiana, which are naturally infected with *M. leprae* were found to harbor the European/North African SNP type 3 strain, indicating that they were infected from human sources (Monot M, Honoré N, Garnier T, Araoz R, Coppee JY, et al. (2005) On the origin of leprosy. Science 308: 1040–1042).

**We have added a sentence to our Discussion mentioning that “The complexities of human-armadillo interaction in relation to M. leprae include the suggestion that transmission can occur in the opposite direction”, citing Monot *et al.* as suggested by the reviewer.**

22. Reviewer LK: An important recommendation raised by the authors is to standardize methods with a protocol based on best practice in specimen collection and simpler and more rapid methods such blood testing for *M. leprae*, rather than taking tissue samples from sacrificed animals. However such tests have lower sensitivity. I am sympathetic with this proposal since armadillos are animals declining in the wild and efforts should be made to preserve them. However, comparability with other studies would be much more complex or even impossible.

**We did mention that the lower sensitivities of rapid methods “might be outweighed by their ease of use and non-lethality” (we changed “are outweighed” to “might be”) but we have added the reviewer’s remark that “As with PCR, these methods would need to be standardized to ensure comparability between studies.”**

23. Reviewer LK: A paper reporting a study of human-armadillo interaction among residents of the State of Ceará, in northeastern Brazil, shows a great variety of contact with armadillos either through hunting, through cooking, or through consumption of armadillo meat. This study raises important and relatively unreported issues about contact between human beings and armadillos that the authors could cite. The authors demonstrate evidence of numerous situations in which leprosy transmission via armadillo is possible (Kerr et al. *Human–armadillo interaction in Ceará, Brazil: Potential for transmission of Mycobacterium leprae*. Acta Tropica. 152: 74-79, Dec 2015).

**We thank the reviewer for recommending this citation which we have appended, with wording suggested by the reviewer, to our discussion where we reported previously unpublished data on armadillo meat consumption habits in Espírito Santo state.**

24. Figure Files: While revising your submission, please upload your figure files to the Preflight Analysis and Conversion Engine (PACE) digital diagnostic tool, <https://pacev2.apexcovantage.com>. PACE helps ensure that figures meet PLOS requirements. To use PACE, you must first register as a user. Then, login and navigate to the UPLOAD tab, where you will find detailed instructions on how to use the tool. If you encounter any issues or have any questions when using PACE, please email us at figures@plos.org.

**We have uploaded all images to PACE.**

25. Data Requirements: Please note that, as a condition of publication, PLOS' data policy requires that you make available all data used to draw the conclusions outlined in your manuscript. Data must be deposited in an appropriate repository, included within the body of the manuscript, or uploaded as supporting information. This includes all numerical values that were used to generate graphs, histograms etc.

**We provide all of the extracted data in a Supplementary File.**

26. Reproducibility: To enhance the reproducibility of your results, PLOS recommends that you deposit laboratory protocols in protocols.io, where a protocol can be assigned its own identifier (DOI) such that it can be cited independently in the future.

**The review protocol was registered with PROSPERO (CRD42019155277) and we provide details of searches in a Supplementary File.**
